# Association of the time in targeted blood glucose range of 3.9–10 mmol/L with the mortality of critically ill patients with or without diabetes

**DOI:** 10.1016/j.heliyon.2023.e13662

**Published:** 2023-02-11

**Authors:** Guo Yu, Haoming Ma, Weitao Lv, Peiru Zhou, Cuiqing Liu

**Affiliations:** a School of Nursing, Jinan University, No. 601, West Huangpu Avenue, Tianhe District, Guangzhou City, Guangdong Province, China; b Health Management Centre, The Fifth Affiliated Hospital of Jinan, South Yingke Avenue, Jiangdong New District, Heyuan City, Guangdong Province, China; c Division of Critical Care, The First Affiliated Hospital of Jinan, No. 613, West Huangpu Avenue, Tianhe District, Guangzhou City, Guangdong Province, China

**Keywords:** Severe disease, Diabetes, Blood glucose control, Time in targeted blood glucose range, Glycemic variability, Mortality

## Abstract

**Purpose:**

The relationship between the TIR and mortality may be influenced by the presence of diabetes and other glycemic indicators. The purpose of this study was to investigate the relationship between TIR and in-hospital mortality in diabetic and non-diabetic patients in ICU.

**Methods:**

A total of 998 patients with severe diseases in the ICU were selected for this retrospective analysis. The TIR is defined as the percentage of time spent in the target blood glucose range of 3.9–10.0 mmol/L within 24 h. The relationship between TIR and in-hospital mortality in diabetic and non-diabetic patients was analyzed. The effect of glycemic variability was also analyzed.

**Results:**

The binary logistic regression model showed that there was a significant association between the TIR and the in-hospital death of severely ill non-diabetic patients. Furthermore, TIR≥70% was significantly associated with in-hospital death (OR = 0.581, P = 0.003). The study found that the coefficient of variation (CV) was significantly associated with the mortality of severely ill diabetic patients (OR = 1.042, P = 0.027).

**Conclusions:**

Both diabetic and non-diabetic critically ill patients should control blood glucose fluctuations and maintain blood glucose levels within the target range, it may be beneficial in reducing mortality.

## Highlights

• The TIR of non-diabetic patients during ICU hospitalization was associated with in-hospital death.
• Diabetic patients seem to have higher blood glucose fluctuations and can tolerate a large TIR range.
• Both diabetic and non-diabetic critically ill patients whose blood glucose levels within the target range have low mortality.

## 1 Introduction

Severely ill patients are more likely to have stress-related hyperglycemia (SHG), large fluctuations in blood glucose levels, and hypoglycemia [1]. Increasing evidence shows that abnormal blood glucose indicators may be associated with morbidity and mortality. In the past two decades, the glucose management of patients in the ICU has been a research hotspot. Intensive insulin therapy in the ICU can be traced back to Leuven's study [2], which showed that the strict control of blood glucose levels with insulin infusion in surgical ICU patients could reduce the mortality of patients. However, recent studies have confirmed that intensive blood glucose control for critically ill patients in the ICU cannot reduce short-term mortality and will increase the incidence of hypoglycemia. Hypoglycemia is an independent risk factor for death in critically ill patients [[3], [4], [5], [6], [7]]. The current professional guidelines still recommend the use of a wider range of blood glucose levels for severely ill adult patients [8,9]. Researchers have been attempted to identify an optimal range of blood glucose levels for critically ill patients in the ICU and reliable indicators for measuring blood glucose levels. The use of only blood glucose or glycosylated hemoglobin as an indicator to measure the patient's blood glucose has certain clinical limitations. These indicators cannot fully reflect the quality of blood glucose control. In addition to blood glucose level indicators, glycemic variability and time in targeted blood glucose range (TIR) may also be important factors that affect the prognosis of critically ill patients. The TIR may be regarded as a “comprehensive” measure that reflects blood glucose control.] The latest diabetes guidelines of the American Diabetes Association (ADA) recommend that for patients with type 1 or type 2 diabetes, the TIR should be greater than 70% at 3.9–7.0 mmol\L, and the proportion of the time below 3.9 mmol\L should be less than 4% [8]. The TIR was initially used for critically ill patients in the ICU. Many researchers have investigated the correlation between TIR level and patient benefit in severely ill patients [[10], [11], [12], [13], [14]].

At present, the relationship between the TIR and prognosis or complications still needs to be investigated using actual clinical data to clarify its role in the evaluation of blood glucose control. Therefore, we retrospectively examined critically ill patients in the ICU to determine the effect of different TIR on the mortality of critically ill patients with diabetes and non-diabetic patients.

## 2 Material and methods

### 2.1 Patients and setting

A retrospective analysis was conducted on 998 severely ill patients with diabetes and without diabetes who were admitted to the ICU of the First Affiliated Hospital of Jinan University from January 1, 2019 to December 31, 2020, which consisted of 250 diabetic patients (DM group) and 748 non-diabetic patients (ND group). All patients were older than 18 years and had been treated in the ICU for at least 1 day. Exclusion criteria were: (1) incomplete death data; (2) in the ICU for less than 24 h; (3) less than 6 blood glucose measurements on the first day after admission; (4) family members gave up treatment; (5) hospital stay exceeded 120 days. The diagnostic criteria for diabetes were based on the 1999 World Health Organization criteria and were determined using the information provided when entering the ICU. Due to the critical and fluctuating conditions of patients with severe diseases, oral glucose tolerance test and HbA1c test were not available; thus, it was not possible to evaluate and diagnose underlying diabetes, and no differentiation was made between type 1 diabetes and type 2 diabetes. The patient's information was extracted from the ICU information system and the hospital's electronic medical record system. The collected information included demographic information, patient diagnosis and comorbidities, past medical history, disease severity score (APACHE II score), ICU hospital stay, blood glucose, hemodialysis days, ventilator-assisted time, other biochemical indicators, and patient outcomes. The ICU information data system does not contain detailed information on the dosage and duration of insulin used by patients; thus, this information was not included in our study. Nutritional support therapy was guided by a standardized program that emphasizes early enteral feeding. During ICU hospitalization, around 10–15% of patients received complete enteral nutrition, and the nutritional support status of these patients was not included in the ICU information data system; thus, it could not be analyzed in this study.

### 2.2 Glucose control

Blood glucose was measured at least every 4 h after admission to the ICU and no less than 6 times per day. Capillary blood glucose was measured using Abbott's FreeStyle Optium Blood Glucose and Ketone Monitoring System (Abbott Diabetes Care, Oxon, UK). Patients receiving continuous intravenous insulin had their blood glucose measured hourly or more often as required. Blood glucose levels during ICU hospitalization were recorded, and Microsoft Excel software was used to calculate the TIR, coefficient of variation (CV), mean blood glucose (MBG), and standard deviation (SD) for each patient. The TIR is defined as the percentage of time spent in the target blood glucose range of 3.9–10.0 mmol/L within 24 h [8]. Glycemic variability is expressed as the CV of blood glucose. The CV is defined as the standard deviation of blood glucose divided by the corresponding mean blood glucose (SD\MBG). According to the guidelines of the ADA in 2014, the blood glucose target range for critically ill patients is 3.9–10.0 mmol/L [15]. A common blood sugar control goal and a uniform insulin infusion standard to maintain blood sugar within the target range were adopted for the patients. Nutritional support standards were managed uniformly according to the ESPEN/ASPEN guidelines [16,17].

## 3 Metrics and statistical methods

Continuous data are expressed as the interquartile range and standard deviation. Differences between groups for normality measurement data were compared by *t*-test, and differences between groups for non-normality measurement data were compared by Mann-Whitney rank sum test. Count data are expressed as percentages. Chi-square test was used for comparison between groups, and Bonferroni test was used for pairwise comparison between groups of three or more. The data of three or more groups were analyzed by ANOVA or non-parametric test, and SNK-Q test was used for further multiple comparisons between two groups.

Univariate regression analysis was used to determine the association between each factor and hospital survival status in the ND and DM groups. P < 0.10 was considered statistically significant. The selected variables were used to construct a binary logistic regression model using the input method to identify independent variables associated with death and determine whether there is an independent association between the TIR and in-hospital death. Covariates could be adjusted according to the clinical setting and professional knowledge; for example, age was not statistically significant in the DM model but was included in the final model. Potential multicollinearity between different covariates was quantified by calculating the variance inflation factor (VIF). A VIF of <5 was considered acceptable. Hosmer-Lemeshow test was used to evaluate the calibration of the model. A P value of <0.05 (two-tailed) was considered statistically significant. Statistical analyses were performed using SPSS software version 17.0 (SPSS Inc., Chicago, IL).

This study was approved by the IRB of the First Affiliated Hospital of Jinan University with the approval document no. KYk-2021-011.

## 4 Results

### 4.1 Clinical characteristics of ICU patients with and without diabetes mellitus

Among the 998 patients, 748 patients were non-diabetic (ND group), and 250 patients were diabetic (DM group). See Table 1 for detailed data. The mean age of the ND group was 60.36 ± 18.64 years, which was lower than that of the DM group (68.55 ± 13.56 years) with statistical significance (P < 0.001). In comparison with patients without diabetes, patients with diabetes had a higher APACHE II score; however, there was no significant difference in the length of ICU stay, length of hospital stay, or mortality. In comparison with the ND group, the DM group had higher blood glucose levels and glycemic variability (all P < 0.001). The probability of hyperglycemia (98.8% vs. 86.2%, P < 0.001) and hypoglycemia (26.7% vs. 19.8%, p = 0.023) was significantly higher in the DM group than in the ND group.

**Table 1:** Baseline characteristics of the ND group and DM group.^a^

Variables	ND group (n = 748)	DM group (n = 250)	P value
Age (years)	60.36 ± 18.64	68.55 ± 13.56	<0.001
Sex: male (%)	65.1	61.9	0.3588
HOSP-LOS (IQR)	15 (8,28)	16 (9, 28)	0.431
ICU-LOS (IQR)	5 (2, 11)	6 (2, 12)	0.068
APACHE II score	25.06 ± 9.24	27.76 ± 9.07	<0.001
Mechanical ventilation (%)	66.2	60.7	0.116
Hemodialysis (%)	21.1	24.2	0.306
Use of vasoactive drugs (%)	64.9	64.4	0.894
Insulin infusion therapy (%)	33.0	56.9	<0.001
Use of corticoids (%)	42.7	31.4	0.0019
Mortality (%)	33.9	40.0	0.08
MBG	9.57 ± 2.42	12.49 ± 2.65	<0.001
SD	4.91 ± 4.79	3.50 ± 1.38	<0.001
CV	24.14 ± 11.71	27.63 ± 8.56	<0.001
TIR	63.40 ± 28.95	32.07 ± 24.46	<0.001
Hypo <3.9 (%)	19.8	26.7	0.023
Hyper >10 (%)	86.2	98.8	<0.001
Hypertension (%)	33.3	66.3	<0.001
CHD (%)	8.8	23.4	<0.001
Stroke (%)	7.9	16.7	<0.001
Hepatitis (%)	3.6	4.4	0.587

a Data are the mean ± SD or percentage unless otherwise indicated. HOSP-LOS, Length of stay in hospital; ICU-LOS: Length of stay in ICU; APACHE, Acute Physiology and Chronic Health Evaluation; IQR, Interquartile range; CV, Coefficient of variation; DM, Diabetes mellitus; ND, Non-diabetic; MBG, Mean blood glucose; SD, Standard deviation; TIR, Time in targeted blood glucose range; Hypo, Hypoglycemia; Hyper, Hyperglycemia; CHD, Coronary heart disease.

### 4.2 Clinical characteristics of the DM and ND groups according to the median TIR

The patients were stratified according to the median TIR of the ND and DM groups. Patients in the ND group whose TIR was lower than the median was divided into the ND-below group (370 patients), those whose TIR was higher than the median was divided into the ND-above group (378 patients), and the DM group was also divided into the DM-below (125 patients) and DM-above (125 patients) groups. See Table 2 for demographic data, clinical parameters, and blood glucose indicators. In the ND group, compared with patients with high TIR, patients with low TIR were older, with a higher APACHE II score, a greater total number of hospitalization days, a higher ventilator usage rate, and a higher mortality rate (all P < 0.005). The MBG (11.22 ± 2.24 vs. 7.89 ± 1.05, P < 0.001) and glycemic variability (27.87 ± 9.02 vs. 20.34 ± 11.57, P < 0.001) were significantly higher among patients with low TIR compared with those with high TIR in the ND group. In addition, low TIR was likely to be accompanied by hyperglycemia (99.47% vs. 72.70%, P < 0.001) but less hypoglycemia (23.1% vs. 43.5%, P < 0.001).

**Table 2:** Clinical characteristics and glucose metrics of diabetic and non-diabetic patients according to the median TIR.^a^

	Below the median	Above the median	P
**ND group**			
**Clinical parameters**			
Sex, male (%)	64.99%	65.40%	0.904
Age (years)	62.51 ± 17.59	58.21 ± 19.45	0.002
ICU-LOS (IQR)	5 (2, 10)	4 (2, 11)	0.161
HOSP-LOS (IQR)	14 (7, 27)	17 (10, 29)	0.039
Mechanical ventilation (%)	73.7%	56.5%	<0.001
Hemodialysis (%)	10.3%	11.9%	0.501
APACHE II score	27.46 ± 9.02	22.59 ± 8.82	<0.001
Mortality (%)	43.8%	24.1%	<0.001
Use of vasoactive drugs (%)	61.4%	63.8%	0.4967
Insulin infusion therapy (%)	28.4%	35.2%	0.0121
Use of corticoids (%)	39.5%	42.9%	0.3452
**Glucose metrics**			
TIR	39.12 ± 19.62	88.14 ± 9.42	<0.001
MBG	11.22 ± 2.24	7.89 ± 1.05	<0.001
SD	3.11 ± 1.26	1.66 ± 1.69	<0.001
CV	27.87 ± 9.02	20.34 ± 11.57	<0.001
Hypo <3.9 (%)	23.1%	43.5%	<0.001
Hyper >10.0 (%)	99.47%	72.70%	<0.001
**DM group**			
**Clinical parameters**			
Sex, male (%)	56.8%	67.2%	0.09
Age (years)	67.19 ± 14.73	69.60 ± 12.09	0.159
ICU-LOS (IQR)	6 (3,12)	5 (2, 12)	0.435
HOSP-LOS (IQR)	16 (9, 27.5)	17 (7.25, 28.75)	0.852
Mechanical ventilation (%)	62.4%	58.4%	0.518
Hemodialysis (%)	22.4%	22.4%	1.000
APACHE II score	27.45 ± 9.01	27.96 ± 9.18	0.662
Mortality (%)	40.8%	39.2%	0.796
Use of vasoactive drugs (%)	46.4%	52.8%	0.3116
Insulin infusion therapy (%)	49.6%	59.2%	0.1276
Use of corticoids (%)	25.6%	34.4%	0.1290
**Glucose metrics**			
TIR	7.46 ± 4.12	50.61 ± 21.51	<0.001
MBG	14.36 ± 2.02	10.60 ± 1.70	<0.001
SD	3.93 ± 1.30	3.07 ± 1.33	<0.001
CV	27.05 ± 7.04	28.26 ± 9.87	0.264
Hypo <3.9 (%)	9.6%	24%	0.002
Hyper >10.0 (%)	100%	97.6%	0.245

a Data are the mean ± SD or percentage unless otherwise indicated. HOSP-LOS, Length of stay in hospital; ICU-LOS: Length of stay in ICU; APACHE, Acute Physiology and Chronic Health Evaluation; IQR, Interquartile range; CV, Coefficient of variation; DM, Diabetes mellitus; ND, Non-diabetic; MBG, Mean blood glucose; SD, Standard deviation; TIR, Time in targeted blood glucose range; Hypo, Hypoglycemia; Hyper, Hyperglycemia.

In the DM group, there was no significant difference in the APACHE II score, length of hospitalization, and mortality between the two TIR subgroups. However, the MBG of patients with low TIR was higher than that of patients with high TIR (14.36 ± 2.02 vs. 10.60 ± 1.70, P < 0.001), and hypoglycemia was less frequent among patients with high TIR (9.6% vs. 24%, P = 0.002).

### 4.3 Association of the TIR with in-hospital death among non-diabetic critically ill patients

Univariate regression analysis showed that when the TIR was a continuous variable, there was a significant association between the TIR and in-hospital death in the ND group (P < 0.001) but not the DM group. In the binary logistic regression model for the ND group (Table 3), the TIR as a continuous variable was significantly associated with in-hospital death after adjustment for age, APACHE II score, mechanical ventilation, and hypoglycemia (OR = 0.991, 95% CI: 0.985–0.997, P = 0.015). As a classification variable, TIR≥70% was significantly associated with mortality (OR = 0.581, 95% CI: 0.405–0.833, P = 0.003). Specifically, TIR≥70% was a protective factor in the absence of in-hospital death. After further adjustment for the CV, the association between the TIR and mortality was weakened, however, there was still a significant association between TIR≥70% and mortality during hospitalization among severely ill non-diabetic patients (OR = 0.628, 95% CI: 0.431–0.915, P = 0.015).

**Table 3:** Logistic regression results of the ND group.

		OR	95% CI		P value
			Lower	Upper	
**TIR as a continuous variable**					
	**Model 1**				
	Age	1.007	0.997	1.017	0.163
	APACHE II score	1.095	1.070	1.121	<0.001
	Mechanical ventilation	1.047	1.016	1.079	0.003
	Hypoglycemia	4.254	2.382	5.529	<0.001
	TIR	0.991	0.985	0.997	0.015
	**Model 2**				
	Age	1.007	0.997	1.017	0.165
	APACHE II score	1.091	1.066	1.117	<0.001
	Mechanical ventilation	1.046	1.015	1.079	0.003
	Hypoglycemia	4.138	2.066	6.117	<0.001
	TIR	0.992	0.986	0.999	0.021
	CV	1.015	0.997	1.034	0.111
**TIR as the classification variable**					
	**Model 3**				
	Age	1.007	0.997	1.017	0.180
	APACHE II score	1.094	1.069	1.120	<0.001
	Hypoglycemia	4.654	3.019	7.176	<0.001
	Mechanical ventilation	1.048	1.017	1.080	0.003
	TIR≥70%	0.581	0.405	0.833	0.003
	**Model 4**				
	Age	1.007	0.997	1.017	0.180
	APACHE II	1.091	1.065	1.116	<0.001
	Hypoglycemia	4.020	2.513	6.430	<0.001
	Mechanical ventilation	1.047	1.016	1.080	0.003
	TIR≥70%	0.628	0.431	0.915	0.015
	CV	1.014	0.997	1.033	0.114
**Include CV but not TIR**					
	Age	1.007	0.997	1.017	0.157
	APACHE II	1.094	1.069	1.120	<0.001
	Hypoglycemia	3.698	2.297	5.953	<0.001
	Mechanical ventilation	1.048	1.017	1.081	0.003
	CV	1.022	1.002	1.042	0.032

CI, Confidence interval; OR: Odds ratio; APACHE, Acute Physiology and Chronic Health Evaluation; TIR, Time in targeted blood glucose range; CV, Coefficient of variation.

In the binary logistic regression model for the DM group (Table 4), after eliminating confounding factors such as age, APACHE II score, mechanical ventilation, and hypoglycemia, the TIR was not significantly and independently associated with ICU survival among severely ill diabetic patients; however, the CV was significantly and independently associated with the mortality of severely ill diabetic patients (OR = 1.042, 95% CI: 1.005–1.080, P = 0.027).

**Table 4:** Logistic regression results of the DM group.

		OR	95% CI		P value
			Lower	Upper	
**Model 1**					
Age		1.035	1.010	1.061	0.005
APACHE II score		1.076	1.039	1.114	<0.001
TIR		0.998	0.986	1.010	0.747
Hypoglycemia		1.595	0.759	3.351	0.218
Mechanical ventilation		1.055	0.999	1.114	0.055
**Model 2**					
Age		1.035	1.011	1.061	0.005
APACHE II score		1.076	1.040	1.114	<0.001
Hypoglycemia		0.607	0.286	1.291	0.195
Mechanical ventilation		1.054	0.998	1.113	0.058
TIR≥25%		0.842	0.471	1.505	0.561
**Model 3**					
Age		1.034	1.009	1.059	0.007
Mechanical ventilation		1.057	1.001	1.117	0.048
Hypoglycemia		1.248	0.551	2.828	0.595
APACHE II score		1.075	1.038	1.114	<0.001
CV		1.042	1.005	1.080	0.027

CI, Confidence interval; OR: Odds ratio; APACHE, Acute Physiology and Chronic Health Evaluation; TIR, Time in targeted blood glucose range; CV, Coefficient of variation.

## 5 Discussion

In this study, through a retrospective analysis of ICU patients with severe diseases, it was found that there was a significant association between the TIR and nosocomial mortality. Furthermore, severely ill patients with and without diabetes showed a significant difference in the association between the TIR and nosocomial mortality.

The TIR was significantly associated with nosocomial mortality in the non-diabetic population; although this association appeared to be weakened after controlling for glycemic variability, it remained significant. Among non-diabetic patients with severe diseases whose TIR was <70%, the mortality rate was nearly twice that of patients with severe diseases whose TIR was ≥70%.

There was no significant association between the TIR and in-hospital death of severely ill patients with diabetes. Although the ICU adopted a unified blood glucose control standard and insulin infusion plan for diabetic and non-diabetic critically ill patients, diabetic patients still had a higher MBG, lower TIR, and higher glycemic variability. In addition, almost all diabetic patients had hyperglycemia, and the incidence of hypoglycemia was higher than that of the ND group. In contrast to the ND group, the TIR was not an independent factor affecting the death of severely ill diabetic patients.

The incidence of hypoglycemia (<3.9 mmol\L) in the DM group was higher than that in the ND group (26.7% vs. 19.8%). Regression analysis showed that non-diabetic patients should minimize the occurrence of hypoglycemia to reduce mortality. The findings of this study are similar to those of previous studies [[18], [19], [20], [21]]. However, there was no significant association between the occurrence of hypoglycemia in diabetic patients and hospital mortality, and diabetic patients appeared to have a stronger tolerance to hypoglycemia. This result are similar to those earlier studies [[5], [6], [7],^11^,[22], [23], [24]].

Severe patients have different degrees of insulin resistance due to stress. In addition, due to abnormal eating, nutrient absorption and utilization disorders, the participation of a variety of hormones and inflammatory factors, and the use of exogenous insulin, the imbalance of physiological buffer system leads to difficulties in the steady regulation of blood glucose, and the overall blood glucose level increases with increased fluctuation range [25]. Previous studies have found that higher glycemic variability has no significant effect on the mortality of severely ill diabetic patients [21]. Severely ill diabetic patients may be more tolerant of lower TIR and higher glycemic variability, as observed in previous studies [23,26,27]. Notably, consistent with other studies, this study found that the state of diabetes may be an important factor affecting the blood glucose indicators of critically ill patients [23,24,28,29]. However, further controlled studies are needed to confirm whether different glucose control regimens should be used for patients with or without diabetes.

This study has several strengths. First, for patients in the ICU, blood glucose was measured throughout the ICU stay, and the number of blood glucose measurements was not less than 6 times a day, which can reduce errors. Second, we used APACHE II to adjust for the effect of disease severity on hospital mortality. APACHE II is widely considered to reflect the severity of the patient's disease and is a good predictor of the death of severely ill ICU patients [30,31]. Moreover, Although the TIR has been previously used for patients with severe diseases, this study may provide some insights into clinical blood glucose management in cases of severe diseases.

This study has some limitations. First, the data were derived from the ICU of a single hospital; thus, the generalization of the results is limited. Second, because the study was an observational study, no evidence of a causal relationship could be drawn between the above glycemic control measures and hospital mortality. Third, the TIR of 3.9–10.0 mmol\L was based on previously published studies. Therefore, the optimal range of blood glucose levels remains unclear. In the future, more extensive and in-depth studies are needed to determine the appropriate range of blood glucose levels for the ICU population. Finally, as with other ICU studies, we can't tell whether ICU patients have type 1 or type 2 diabetes. Patients admitted to the ICU are rarely tested for HbA1c, making it impossible to evaluate the patients' previous glycemic control, which results in a substantial limitation of the study.

Overall, the ICU patient data in this study demonstrated a strong association between the TIR and mortality risk, especially among non-diabetic patients with severe diseases. The TIR is easy to measure and evaluate, and as a variable to measure the effect of blood glucose control in the short term, it is significant in the ICU context and may be used as a quality indicator to guide ICU patients who require critical care. The findings of this study may also be used as a reference for the design of intensive insulin trials for critically ill patients in the future.

## 6 Conclusions

This study showed that TIR≥70% was associated with reduced mortality among critically ill patients without diabetes, however, this association was not seen in people with diabetes. Meanwhile, the CV was significantly and independently associated with the mortality of severely ill diabetic patients. Both diabetic and non-diabetic critically ill patients should control blood glucose fluctuations and maintain blood glucose levels within the target range, it may be beneficial in reducing mortality. The findings may provide novel ideas for the clinical glycemic control of critically ill patients and important information for further studies on the relationship between the glycemic indices and mortality of ICU patients.

## Declarations

This manuscript has not been published or presented elsewhere in part or in entirety, and is not under consideration by another journal. There are no conflicts of interest to declare. All authors have contributed to the creation of this manuscript for important intellectual content and read and approved the final manuscript.

## Ethics approval and consent to participate

This study was approved by the IRB of the First Affiliated Hospital of Jinan University with the approval document no. KYk-2021-011. All study data were maintained anonymously.

## Availability of data and materials

The datasets used and analyzed during the current study are available from the corresponding author on reasonable request.

## Author contribution statement

Peiru Zhou: Conceived and designed the experiments; Analyzed and interpreted the data; Contributed reagents, materials, analysis tools or data. Guo Yu; Haoming Ma: Conceived and designed the experiments; Performed the experiments; Analyzed and interpreted the data; Wrote the paper. Cuiqing Liu: Contributed reagents, materials, analysis tools or data. Weitao Lv: Conceived and designed the experiments; Contributed reagents, materials, analysis tools or data.

## Funding statement

This work was supported by 10.13039/501100007162Department of Science and Technology of Guangdong Province Plan Project “Diabetes Intelligent Wearable Monitoring Device and Complication Prevention and Control Cloud Platform” [2016B010108008].

## Data availability statement

Data will be made available on request.

## Declaration of interest's statement

The authors declare no conflict of interest.

## Additional information

No additional information is available for this paper.
